# Targeting immune checkpoints in hematological malignancies

**DOI:** 10.1186/s13045-020-00947-6

**Published:** 2020-08-12

**Authors:** Basit Salik, Mark J. Smyth, Kyohei Nakamura

**Affiliations:** Immunology in Cancer and Infection Laboratory, QIMR Berghofer Medical Research Institute, 300 Herston Road, Herston, Queensland 4006 Australia

**Keywords:** Immunotherapy, Immune checkpoint molecule, Tumor microenvironment, Hematological malignancy

## Abstract

Immune checkpoint blockade (ICB) therapies such as anti-programmed death 1 (PD-1) and anti-CTLA-4 (cytotoxic T lymphocyte-associated protein 4) have dramatically transformed treatment in solid tumor oncology. While immunotherapeutic approaches such as stem cell transplantation and anti-cancer monoclonal antibodies have made critical contributions to improve outcomes in hematological malignancies, clinical benefits of ICB are observed in only limited tumor types that are particularly characterized by a high infiltration of immune cells. Importantly, even patients that initially respond to ICB are unable to achieve long-term disease control using these therapies. Indeed, primary and acquired resistance mechanisms are differentially orchestrated in hematological malignancies depending on tumor types and/or genotypes, and thus, an in-depth understanding of the disease-specific immune microenvironments will be essential in improving efficacy. In addition to PD-1 and CTLA-4, various T cell immune checkpoint molecules have been characterized that regulate T cell responses in a non-redundant manner. Several lines of evidence suggest that these T cell checkpoint molecules might play unique roles in hematological malignancies, highlighting their potential as therapeutic targets. Targeting innate checkpoint molecules on natural killer cells and/or macrophages has also emerged as a rational approach against tumors that are resistant to T cell-mediated immunity. Given that various monoclonal antibodies against tumor surface proteins have been clinically approved in hematological malignancies, innate checkpoint blockade might play a key role to augment antibody-mediated cellular cytotoxicity and phagocytosis. In this review, we discuss recent advances and emerging roles of immune checkpoint blockade in hematological malignancies.

## Background

Immunotherapy has emerged as a new pillar of cancer treatment. Over the past decade, immune checkpoint blockade (ICB) therapies such as monoclonal antibodies (mAbs) that target cytotoxic T lymphocyte-associated protein 4 (CTLA-4) or the programmed cell death protein 1 (PD-1) pathway have dramatically changed therapeutic strategies in certain types of advanced malignancies. Humanized anti-CTLA-4 antibody, ipilimumab, reportedly doubles 10-year survival rates for metastatic melanoma compared to historical control data, and its FDA approval marked a turning point for immunotherapy [1, 2]. Blockade of PD-1 or its ligand, PD-1 ligand (PD-L1), has displayed superior clinical responses with fewer side effects in a broad range of cancers [3–9].

Historically, immunotherapy has been well-studied in hematological malignancies as supported by the success of stem cell transplantation (SCT) and various mAbs against tumor surface proteins. While newly developed ICB therapies are actively being tested, primary and acquired resistance remain major barriers in utilizing them against a broad range of hematological malignancies. To this end, it is critically important to understand the complex immune regulatory mechanisms mediated by immune checkpoint molecules as well as the disease-specific immune milieu. In addition to therapeutic blockade of PD-1 and CTLA-4, various immune checkpoint molecules that regulate innate and/or adaptive immune responses have emerged as potential therapeutic targets. In this review, we will provide an overview of the basic and clinical aspects of ICB therapy and discuss their potential in harnessing anti-tumor immunity in hematological malignancies.

## Immune regulation by PD-1 and CTLA-4

Over the past decade, various ICB drugs have received FDA approval including anti-CTLA-4 (ipilumumab), anti-PD-1 (pembrolizumab, nivolumab, and cemiplimab), and anti-PD-L1 (atezolizumab, avelumab, and durvalumab). It is appreciated that the interaction between CTLA-4 and its ligands CD80 (B7-1) and CD86 (B7-2) critically regulates T cell priming at the interface between T cells and antigen-presenting cells (APCs), whereas the interaction between PD-1/PD-L1 controls T cell responses at the effector phase [10]. Immune-related adverse events (irAEs) are commonly seen in patients treated with either anti-CTLA-4 or anti-PD-1/PD-L1, but the incidence of severe irAEs (grade 3 or 4) is much more frequent in patients treated with CTLA-4 blockade [11]. It should be noted that even after clinical approval of these therapies, a number of studies have revealed new molecular mechanisms of immune regulation by PD-1 and CTLA-4. Their immune regulatory mechanisms are far more complicated; therefore, an in-depth understanding of the complex interplay will provide new insights into the mechanisms of ICB therapies.

### CTLA-4

**Fig. 1:**
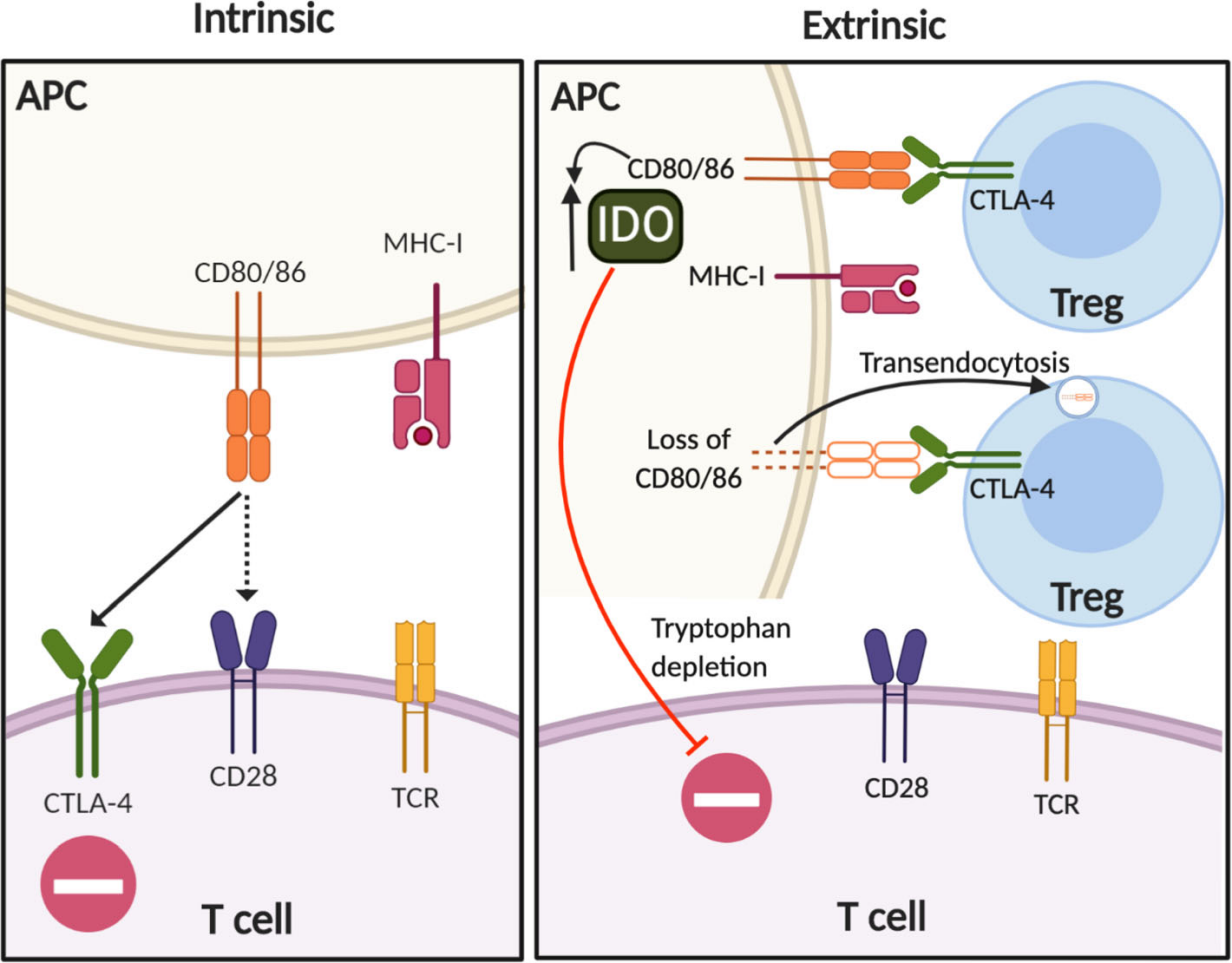
CTLA-4-mediated immune regulation. Schematic illustrating T cell-intrinsic (left) and extrinsic regulation by CTLA-4 (right). Left: CTLA-4 is upregulated on activated T cells and competes with the CD28 co-stimulatory receptor due to its higher affinity for CD80/CD86. Right: CTLA-4 plays a critical role in Treg-mediated immune regulation. The CTLA-4/CD80 interaction between Treg/APCs induces indoleamine 2,3-dioxygenase (IDO), a key enzyme that suppresses T cells by tryptophan deprivation. Additionally, Tregs down-modulate CD80/86 expression on APCs by transendocytosis

CTLA-4 expression and function are intrinsically associated with T cell activation (Fig. 1, left). Upon T cell receptor (TCR) engagement, CTLA-4 is upregulated with peak expression occurring 2 to 3 days after activation [12]. CTLA-4 accumulates at the immunological synapse between T cells and APCs, where CTLA-4 is stabilized by CD80 ligand binding [13, 14]. Due to its higher avidity and affinity for CD80/CD86, CTLA-4 competes with the costimulatory molecule CD28 leading to the negative regulation of activated T cells [15–17].

In addition to cell-intrinsic regulation, CTLA-4 also regulates T cell activation in a cell-extrinsic manner that is primarily mediated by regulatory T cells (Tregs) (Fig. 1, right) [18, 19]. Indeed, Treg-specific deletion of CTLA-4 results in aberrant T cell activation and autoimmunity highlighting CTLA-4 as a key functional molecule for Treg-mediated immune tolerance [20, 21]. Of note, extrinsic regulation by CTLA-4 cannot be simply explained by the competitive inhibition of the interaction between CD28 and CD80/86 at the immunological synapse. For example, the interaction between CTLA-4 and CD80/86 upregulates indoleamine 2,3-dioxygenase (IDO) in DCs, a key enzyme for tryptophan catabolism [22]. Thus, the interaction between Tregs and DCs limits antigen-specific T cell responses by IDO-mediated tryptophan depletion.

Another regulatory mechanism of CTLA-4 is its ability to rapidly capture CD80/CD86 from APCs by a process of trans-endocytosis [23]. Although both effector lymphocytes and Tregs are known to have the ability to mediate CTLA-4-dependent deprivation of CD80/CD86 from APCs [24], Ovcinnikovs et al. recently demonstrated that Tregs, rather than activated conventional T cells, are predominantly responsible for the trans-endocytosis of CD80/CD86 in vivo [25]. They also showed that migratory DCs, rather than tissue resident DCs in lymph nodes, are the major target for CTLA-4-dependent deprivation of CD80/CD86, providing an important mechanistic insight into anti-CTLA-4 therapy [25]. While the CTLA-4-dependent trans-endocytosis of CD80/86 and subsequent degradation of these ligands contribute to immune tolerance, CTLA-4 itself is constitutively internalized in a ligand-independent manner, undergoing both recycling and degradation in activated T cells [26]. Recently, Lo et al. provided evidence that optimal recycling of CTLA-4 is critical for maintaining immune tolerance [27]. It is reported that patients with loss-of-function of the *LRBA* gene (encoding the lipopolysaccharide-responsive and beige-like anchor protein) develop early-onset autoimmunity and lymphoproliferative disease, a similar syndrome seen in patients with IPEX (immunodysregulation polyendocrinopathy enteropathy X-linked) syndrome caused by *FOXP3* mutations [28]. Lo et al. showed that LRBA co-localizes with CTLA-4 in recycling endosomes and that LRBA deficiency accelerates CTLA-4 turnover leading to degradation in lysosomes [27]. The balance between CTLA-4 recycling and degradation provides an important implication for therapeutic strategies targeting CTLA-4. Indeed, immune-related adverse events (irAEs) remain as a major barrier in the therapeutic targeting of CTLA-4, occurring in 60–65% of patients treated with ipilimumab [29]. Zhang et al. showed that irAE-prone anti-CTLA-4 mAbs (including ipilimumab) rapidly direct surface CTLA-4 for lysosomal degradation by preventing binding of CTLA-4 to LRBA. In contrast, engineered anti-CTLA-4 mAbs that dissociate from CTLA-4 in response to low pH in endosomal vesicles allow CTLA-4 to be recycled in an LRBA-dependent manner. Strikingly, these novel pH-sensitive anti-CTLA-4 mAbs prevent irAEs with an enhanced preclinical anti-tumor efficacy [30]. Thus, CTLA-4 recycling should be an important consideration for CTLA-4 blockade.

### PD-1

Like CTLA-4, PD-1 also plays a critical role for regulating T cell activation and maintenance of peripheral tolerance [31–33]. Upon engagement of its ligands PD-L1 or PD-L2 during antigen stimulation, PD-1 becomes clustered with the TCR and subsequently recruits the tyrosine phosphatase SHP2 to its cytoplasmic domain [34]. By analyzing the direct targets of PD-1-bound phosphatase(s), Hui et al. recently showed that CD28 signaling is the most sensitive target for PD-1-SHP2-mediated dephosphorylation, while only a part of the TCR signaling components undergo dephosphorylation [35]. Another independent group also demonstrated that CD28 co-simulation is indispensable for optimal CD8 T cell responses against tumors and viral infections by PD-1 blockade [36]. These findings highlight CD28 signaling as a key target of PD-1-mediated immune regulation. Of note, PD-1 also transcriptionally regulates T cell activation by suppressing genes induced by TCR activation [37]. Specifically, genes induced by a strong TCR signal (including genes encoding cytokines and effector molecules) are highly sensitive to PD-1-mediated repression whereas genes that are efficiently induced by TCR stimulation (e.g., genes related to cell survival and cell signaling) show resistance [37]. Thus, in addition to the PD-1/SHP2-mediated dephosphorylation of CD28, PD-1 is implicated in transcriptional regulation of TCR-induced effector molecules, highlighting a broad impact of PD-1 on T cell activation.

**Fig. 2:**
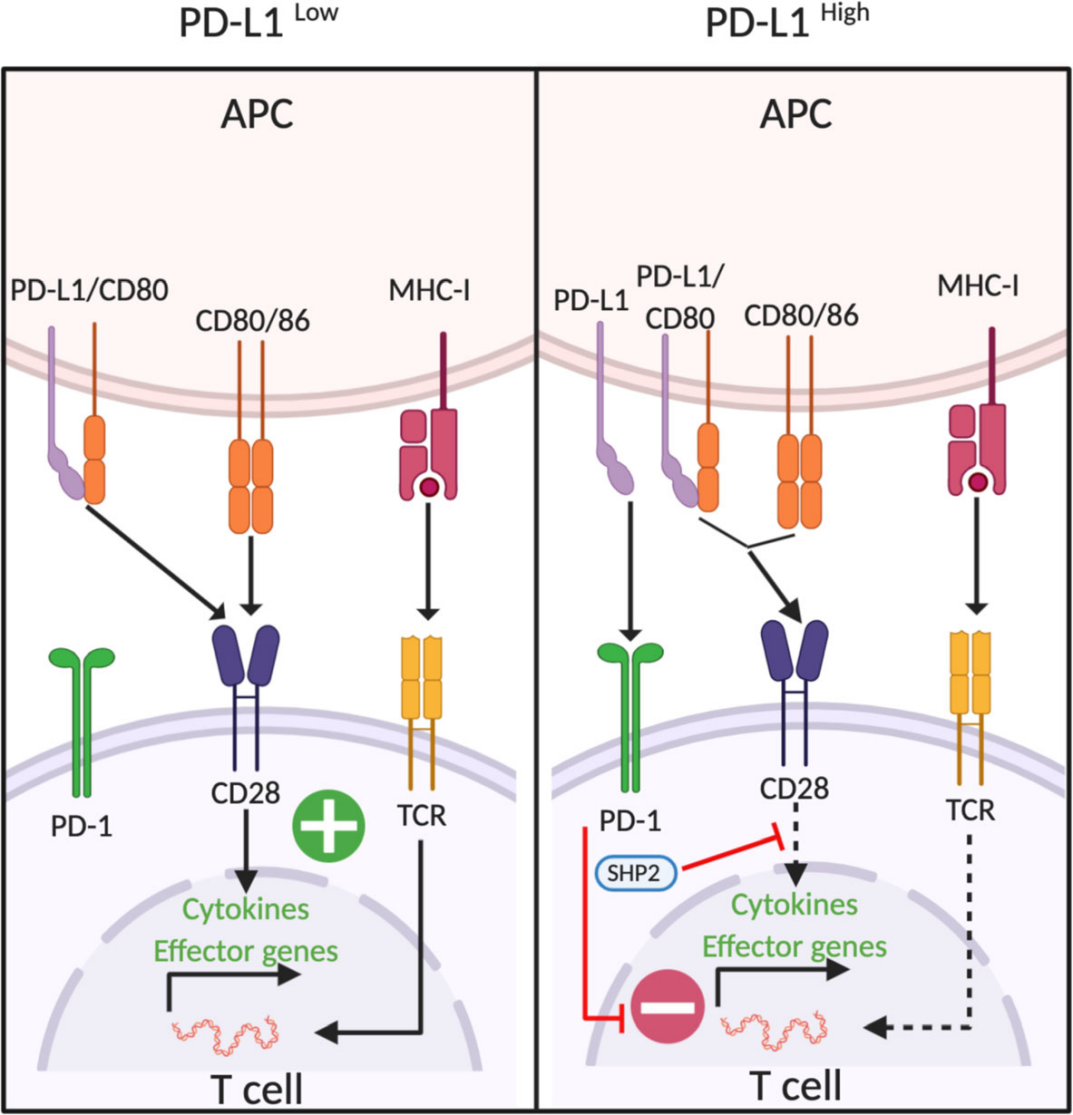
PD-1-mediated immune regulation. Under low expression levels of PD-L1, CD80 restricts PD-L1 function by forming the PD-L1/CD80 *cis*-heterodimer. The PD-L1/CD80 *cis*-heterodimer prevents the PD-1/PD-L1 *trans*-interaction, whereas the ability to bind to the CD28 co-stimulatory receptor is retained (left). Upregulation of PD-L1 on APCs allows the PDL-1/PD-1 *trans*-interaction, leading to SHP2-dependent negative regulation of the CD28 signaling pathway as well as transcriptional repression of TCR-induced effector genes (right)

In addition to the interaction between PD-1 and PD-L1 on T cells and APCs (namely, the PD-1/PD-L1 *trans-*interaction), respectively, PD-L1 interactions *in cis* with PD-1 or CD80 have emerged as important factors for immune modulation. Zhao et al. initially showed that a subset of tumor-infiltrating APCs co-express PD-1 and PD-L1 and that PD-L1/PD-1 *cis* interaction can prevent PD-L1 binding to T cell intrinsic PD-1 *in trans* [38]. However, given that only a small subset of DCs co-express PD-L1 and PD-1, the significance of this interaction for ICB therapies remains unclear. More recently, several lines of evidence demonstrate that the *cis* interaction between PD-L1 and CD80 is predominantly implicated in immune modulation on APCs [39–41] (Fig. 2). Indeed, Sugiura et al. showed that the CD80/PD-L1 *cis* interaction on dendritic cells (DCs) can impede the PD-L1/PD-1 *trans* binding between DCs and T cells in a competitive manner [40]. Strikingly, gene-modified mice that cannot form the PD-L1/CD80 *cis*-heterodimer ameliorate anti-tumor T cell responses as well as autoimmunity, suggesting that the PD-1/PD-L1 inhibitory pathway is enhanced in the absence of the PD-L1/CD80 *cis*-heterodimer [40]. These findings highlight that CD80 on APCs augments T cell activity not only by CD28-mediated co-stimulatory signals but by also restricting PD-L1 (Fig. 2).

Not surprisingly, the PD-L1/CD80 *cis*-heterodimer also affects CTLA-4-mediated immune regulation. Recently, Zhao et al. showed that the PD-L1/CD80 *cis-*heterodimer prevents the interaction between CTLA-4 and CD80 as well as the subsequent trans-endocytosis of CD80 while allowing the CD80 and CD28 interaction to remain intact [41]. Notably, inhibiting the formation of the PD-L1/CD80 *cis*-heterodimer by anti-PD-L1 mAb led to the loss of CD80 from APCs, whereas anti-PD-L1 mAb in combination with anti-CTLA-4 maintained CD80 expression on APCs [41]. These results suggest that selective blockade of the PD-1/PD-L1 *trans*-interaction, but not the PD-L1/CD80 cis-heterodimer formation, may be an important therapeutic approach. Moreover, soluble forms of PD-L1 and/or exosomal PD-L1 have emerged as immunosuppressive mediators [42, 43]. Yet, it remains to be elucidated how membrane-bound form of PD-L1 and soluble PD-L1 differentially regulate T cell responses.

The complex interplay between the B7 family of ligands (CD80, CD86, and PD-L1) and the CD28 receptor superfamily (CD28, CTLA-4, and PD-1) is implicated in fine-tuning immune responses at the T cell APC interface. Indeed, Lin et al. provided preclinical evidence that PD-L1 expression on APCs is indispensable for tumor control by PD-1/PD-L1 blockade, whereas neither knockout nor overexpression of PD-L1 in tumor cells affects efficacy [44]. Notably however, overexpression of tumor PD-L1 is associated with the clinical efficacy of PD-1/PD-L1 blockade in various hematological malignancies, as discussed in the next section. Moreover, the expression of CD80/CD86, in contrast to solid malignancies, is frequently observed in tumor cells from hematological malignancies (such as B cell lymphoma) [45] raising the possibility that the crosstalk between the B7 ligand family and the CD28 receptor superfamily may be further complicated in hematological malignancies. Altogether, the extent to which individual mechanisms prevail and effect clinical response to ICB may depend on the immune landscape that can be influenced by several factors in the various hematological malignancies.

## Clinical response and resistance to immune checkpoint blockade in hematological malignancies

With a growing understanding of cancer biology, as well as the tumor microenvironment (TME), it is becoming increasingly clear that resistance as well as response to ICB can be strongly influenced by disease-specific factors including the immune landscape of the disease. A number of studies have reported encouraging results in various hematologic malignancies. Some of the basic and clinical aspects (Table 1) of those findings will be the focus of our discussion here.

**Table 1:** Notable clinical trials targeting immune checkpoints in hematological malignancies

Clinical trial	Phase	Patient characteristics	Intervention	Response	Reference
NCT01592370	I	Relapsed or refractory HL	Nivolumab	ORR 87%	[46]
CheckMate 205 (NCT02181738)	II	cHLCohort A: brentuximab vedotin naïve Cohort B: brentuximab vedotin after auto-HCT Cohort C: brentuximab vedotin before and/or after auto-HCT Cohort D: nivolumab monotherapy followed by nivolumab plus doxorubicin, vinblastine, and dacarbazine for newly diagnosed HL	Nivolumab	ORR: Cohort A 65% Cohort B 68% Cohort C 73% Cohort D 84%	[47, 48]
KEYNOTE-013 (NCT01953692)	I	cHL after brentuximab vedotin failure	Pembrolizumab	ORR 65%	[49]
KEYNOTE-087 (NCT02453594)	II	Relapsed or refractory cHL, Cohort 1: after ASCT/brentuximab vedotin Cohort 2: ineligible for ASCT and experienced treatment failure with brentuximab vedotin Cohort 3: No brentuximab vedotin after ASCT	Pembrolizumab	ORR: Cohort 1 73.9% Cohort 2 64.2% Cohort 3 70%	[50]
NCT02038933	II	Relapsed or refractory DLBCL Cohort 1: auto-HCT-failed Cohort 2: auto-HCT-ineligible	Nivolumab	ORR: Cohort 1 10% Cohort 2 3%	[51]
NCT02446457	II	Relapsed FL	Pembrolizumab Rituximab	Pre-planned interim analysis: ORR 80%	[52]
NCT03245021	II	Previously untreated FL	Single-agent nivolumab followed by combined nivolumab and rituximab	Pre-planned interim analysis: ORR 84%	[53]
NCT03278782	I/II	Relapsed or refractory peripheral T cell lymphoma (PTCL)	Pembrolizumab Romidepsin	ORR 44%	[54]
NCT02243579	II	Recurrent mycosis fungoides and Sezary syndrome	Pembrolizumab	ORR 38%	[55]
KEYNOTE-023 (NCT02036502)	I	Relapsed or refractory MM	Pembrolizumab combined with lenalidomide and low-dose dexamethasone	ORR 44%	[56]
KEYNOTE-183 (NCT02576977)	III	Relapsed or refractory MM	Pembrolizumab plus pomalidomide and dexamethasone	Pembrolizumab plus pomalidomide and dexamethasone group: Median PFS: 5.6 months (95% CI 3.7–7.5); Pomalidomide and dexamethasone group: 8.4 months (5.9–not reached)	[57]
KEYNOTE-185 (NCT02579863)	III	Treatment-naive MM	Pembrolizumab plus lenalidomide and dexamethasone	Progression-free survival estimates at 6-months were 82.0% (95% CI 73.2–88.1) versus 85.0% (76.8–90.5; hazard ratio [HR] 1.22; 95% CI 0.67–2.22; *p* = 0.75)	[58]
NCT01822509	I	Patients with relapse after allogeneic transplantation: AML (in 12 patients, including 3 with leukemia cutis and 1 with a myeloid sarcoma), HL (in 7), NHL (in 4), and myelodysplastic syndrome (in 2). One patient each had MM, myeloproliferative neoplasm, and acute lymphoblastic leukemia	Ipilimumab	Patients that received a dose of 10 mg/kg: CR (23%)	[59]
NCT02397720	II	Relapsed or refractory AML	Azacitidine and nivolumab	ORR 33%	[60]

*cHL* classic Hodgkin lymphoma, *NHL* non-Hodgkin’s lymphoma, *PTCL* peripheral T cell lymphoma, *MM* multiple myeloma, *AML* acute myeloid leukemia, *ASCT* allogeneic stem cell transplantation, *HCT* hematopoietic cell transplantation, *ORR* objective response rate, *CR* complete response, *HR* hazard ratio, *PFS* progression-free survival, *FL* follicular lymphoma, *DLBCL* diffuse large B cell lymphoma

### Hodgkin lymphoma

Among various hematological malignancies, therapeutic benefits of PD-1 blockade have been best demonstrated in patients with Hodgkin lymphoma (HL). In an early clinical trial of 23 relapsed or refractory HL patients, nivolumab showed an objective response rate (ORR) of 87%, with a complete response (CR) of 17% [46]. Clinical efficacy and safety profile of nivolumab monotherapy was further demonstrated in the CheckMate 205 trial of 80 HL patients who failed autologous stem-cell transplantation and brentuximab vedotin (an antibody-drug conjugate comprising anti-CD30 mAb conjugated to an anti-microtubule agent) [47, 48, 61]. Another PD-1 mAb, pembrolizumab, also showed similar efficacy against HL in clinical trials such as KEYNOTE-013 [49] and KEYNOTE-087 [50]. These results led to the FDA approval of nivolumab (in 2016) and pembrolizumab (in 2017) for relapsed or refractory HL patients who had failed multiple lines of therapy.

Clinical responsiveness to PD-1 blockade in HL has been explained by multiple unique tumor intrinsic and extrinsic factors. In solid malignancies, high TMB (high TMB; ≥ 20 coding mutations per megabase) has been recognized as an independent predictor for clinical responses to ICB therapies [62]. However, in HL, it is reported that TMB does not correlate with responsiveness [63]. Indeed, Liang et al. investigated TMB in 34 HL patients and found that only 15% of the patients had high TMB [64]. Despite this fact, amplification of 9p24.1 (locus-containing JAK2/PDL1/PDL2), a frequently observed cytogenetic abnormality in classical HL patients, induces aberrant overexpression of PD-L1 on tumor cells [65], suggesting that tumor PD-L1 might dampen anti-tumor immunity. Indeed, Epstein-Barr virus (EBV) infection can also contribute to PD-L1 upregulation [66]. While similar frequencies of 9p24.1 amplification in EBV-related or EBV-unrelated cases have been reported in cHL, EBV-positive cHL cases have been shown to be more likely to have higher PD-L1 expression levels [67]. Additionally, through viral oncoprotein latent membrane protein 1 (LMP1), EBV may also sustain an immune suppressive microenvironment [68]. Thus, it is possible that the presence of these immune suppressive features in EBV-related cHL may allow patients with this particular sub-type to be more susceptible to ICB.

In addition to the tumor-intrinsic overexpression of PD-L1, the immune microenvironment of HL is critically responsible for responsiveness to PD-1 blockade. The TME of classical HL consists of rare (0.1–1%) malignant cells called Hodgkin Reed-Sternberg (HRS) cells and an abundant immune cell infiltrate which is markedly distinct from the TME observed in non-Hodgkin’s lymphoma (NHL) [69]. Thus, the unique immunologically “hot” (or inflamed) TME critically contributes to responsiveness to PD-1 blockade [70]. To understand the immune landscape, Cader et al. recently performed mass cytometry of 7 newly diagnosed classical HL patients [71]. Notably, they found that CD4^+^ T cells especially Th1-polaralized effector cells and Tregs were major T cell subsets in the TME and that MHC-I expression was frequently lost in tumor cells [71]. This result raises the possibility that CD4^+^ T cells, rather than CD8^+^ T cells, may be key players in anti-tumor immunity against HL. Indeed, Roemer et al. also reported that β2-microglobulin (β2M) and MHC-I were not expressed on tumor cells in more than 60% of HL patients and that the expression level of MHC-II and PD-L1 predicts therapeutic response to nivolumab in HL patients [72]. More recently, Patel et al. investigated checkpoint molecules by multiplexed immunofluorescence imaging and observed the expansion of CTLA-4^+^ PD-1^−^ CD4 T cells in close proximity to CD86^+^ tumor cells or tumor-associated macrophages (TAMs) [73]. This suggests that the interaction between CTLA-4 and CD86 might also act as a key negative regulator in HL.

Overall, these results highlight the importance of characterizing the disease-specific TME and T cell phenotypes in order to determine optimal therapeutic targets. Despite the clinical benefits of PD-1 blockade, some HL patients experience recurrence after PD-1 blockade therapy. Currently, the randomized phase 2 study of brentuximab vedotin and nivolumab with or without ipilimumab is ongoing (NCT01896999) to assess the efficacy of dual CTLA-4 and PD-1 blockade. LAG-3 might be a potential target considering the recent single-cell RNA sequence (scRNA-seq) analysis which showed that a subset of HL-associated T cells expressed high levels of this checkpoint molecule [74]. Alternatively, given that loss of MHC-II confers therapeutic resistance to HL, harnessing innate immunity might be a key approach. The PD-1 and PD-L1 interaction is implicated in negative regulation of NK cells and monocytes/macrophages [75]. As discussed later, several innate immune checkpoint inhibitors are being developed. The combination of innate checkpoint inhibitors may improve clinical responses against HL with acquired resistance to T cell-mediated anti-tumor immunity.

### Non-Hodgkin lymphoma

In contrast to HL patients, PD-1 blockade has not shown remarkable clinical responses in patients with NHL such as diffuse large B cell lymphoma (DLBCL) and follicular lymphoma (FL). In a phase 2 clinical trial of nivolumab in relapsed or refractory DLBCL, ORR of monotherapy was 3% and 10% in transplant-ineligible patients and relapsed patients after autologous SCT, respectively [51]. The efficacy of nivolumab and ibrutinib (a Bruton’s tyrosine kinase inhibitor) combination has been evaluated in relapsed or refractory NHL (DLBCL and FL) and chronic lymphocytic leukemia patients. However, overall response of the combination was comparable to that of ibrutinib monotherapy [76], suggesting that PD-1 blockade had limited contribution to disease control. Indeed, PD-1 blockade in combination with rituximab has been shown to be effective in rituximab refractory FL [52]. However, ICB can also be utilized to prime the immune system prior to such tumor-targeted therapies. In fact, this concept was trialed in a phase 2 study in treatment of naïve FL patients using a combination of nivolumab and rituximab [53]. Interim analyses indicate an ORR of 84% with 47% achieving CR, suggesting a favorable toxicity profile along with high overall and complete response rates [53].

Differential responsiveness to PD-1 blockade between DLBCL patients and HL patients can be primarily explained by their different levels of immune infiltration. Indeed, the transcriptional and histological comparison of the immune microenvironment between HL and DLBCL showed an immunologically “cold” (or “non-inflamed”) TME in DLBCL in contrast to the “hot” (or “inflamed”) TME in HL [77]. Of note, in DLBCL patients treated with rituximab-based standard chemo-immunotherapy (R-CHOP), high infiltration of T cells predicts better prognosis, whereas the presence of PD-1^high^ T cells predicts poor prognosis [78, 79] indicating that anti-lymphoma immunity still plays an indispensable role for disease control against immunologically cold DLBCL.

The immunologically “cold” TME is created by tumor-intrinsic factors such as high proliferation rate of lymphoma cells and oncogene-driven immune exclusion [70]. Various factors are reported to be associated with immunologically “cold” TME such as double rearrangements of *MYC* and *BCL2* and/or *BCL6* [80], deletion or mutation of *PTEN* [81], and Epstein-Barr virus-related subtypes [82]. Above all, several lines of evidence suggest that *EZH2* mutations critically contribute to immune exclusion phenotypes. It is reported that MHC-I and MHC-II are lost in 40–60% and 20–40% of DLBCL patients, respectively, and *EZH2* mutations are highly enriched in MHC-deficient subsets of patients [83]. The EZH2-mediated silencing of genes related to MHC-I expression is implicated in immune evasion in a wide range of other malignancies, as Burr et al. also demonstrated this immune evasion mechanism by a whole genome CRISPR/Cas9 screen [84]. Thus, inhibition of EZH2 activity by a small molecule inhibitor might be a potential approach to sensitize tumor cells to T cell-mediated anti-tumor immunity.

As seen in HL, EBV has also been linked to a number of malignant NHLs [85]. In a recent study, Kim et al. reported that more patients with EBV-positive NHL responded to pembrolizumab than EBV-negative subtypes [86]. Moreover, high PD-L1 expression was reported in EBV-positive NHL as compared to EBV-negative NHL [86]. In agreement with this, Kataoka et al. recently reported a high frequency of PD-L1/PD-L2 containing somatic aberrations in various EBV-positive lymphoma subtypes [87]. In addition, a distinct pattern of somatic alterations in EBV-positive DLBCL was reported where the genetic profile was found to be distinct from EBV-negative DLBCL [87]. While ICB in treating virus-associated cancers appears promising, more clinical trials are needed to verify if viral infections such as EBV can effectively predict ICB efficacy.

Another important tumor-intrinsic factor is gene alterations (amplifications or translocations) of *PD-L1* that are observed in ~ 25% of DLBCL patients (especially the non-germinal center type) [88]. This DLBCL subset with aberrantly overexpressed PD-L1 is characterized by high infiltration of clonal T cells and low expression of tumor MHC-I. While patients with this type of DLBCL show inferior progression-free survival following front-line chemo-immunotherapy, they show, strikingly, good responsiveness to PD-1 blockade [88]. Thus, *PD-L1* alterations may be a useful biomarker in predicting responsiveness to PD-1 blockade in DLBCL patients. Still, it remains unknown how we can overcome therapeutic resistance in patients with the immunologically “cold” DLBCL (low T cell infiltration and low MHC expression on tumor cells). Blockade of innate checkpoint molecules in combination with rituximab might be a possible approach to augment NK cell-mediated antibody-dependent cellular cytotoxicity (ADCC) and macrophage-mediated antibody-dependent cellular phagocytosis (ADCP).

NK/T cell lymphomas have a distinct immunophenotype, and PD-1 blockade has shown some efficacy in relapsed or refractory NK/T cell lymphomas [89]. In a recent retrospective study of seven patients with relapsed or refractory NK/T cell lymphomas treated with pembrolizumab at diagnosis, the ORR was reported to be 57.1% with a complete response occurring in 2 patients [90]. Large scale trials are warranted to further assess long-term clinical responses. Notably, modest responses to single agent PD-1 blockade have been observed in peripheral T cell lymphomas (PTCL) [91]. Indeed, mutations in epigenetic modifier genes are often observed in PTCL, and such mutations may promote immune escape. To this end, a recent phase I/II trial combined pembrolizumab and a histone deacetylase inhibitor in relapsed or refractory PTCL patients [54]. From the 15 evaluable patients, 3 were complete responders that remained in remission for at least 10 months [54]. These early findings are encouraging, and therefore, further effort in this direction should be pursued. Furthermore, some responses to ICB have also been reported in patients with common subtypes of cutaneous T cell lymphomas. PD-1 blockade led to an ORR of 38% in patients with mycosis fungoides or Sézary syndrome in a phase 2 trial demonstrating a favorable safety profile and modest anti-tumor activity [55]. Notably, some flare reactions such as worsening of erythema were reported in patients with Sézary syndrome, and such skin flare reactions were found to be associated with high PD-1 expression on circulating Sezary cells before therapy [55]. Although high PD-1 expression may be an effective predictor of this reaction, further validation is warranted. On the contrary, PD-1 blockade led to rapid progression in patients with adult T cell leukemia/lymphoma (ATLL) in a recent study [92]. Rapid clonal expansion of malignant T cells was observed in patients treated with PD-1 blockade, suggesting a tumor-intrinsic regulatory role of PD-1 in ATLL [93]. This is in line with a previous report from a T cell lymphoma mouse model where PD-1 activity enhanced PTEN levels, and PD-1 deletion after an oncogenic insult resulted in aggressive lymphomas in vivo [94]*.* These data highlight the growing need to mechanistically understand the underlying mechanisms of ICB as we attempt to extend their promise to an ever-increasing list of malignancies.

### Multiple myeloma

Multiple myeloma (MM) remains an incurable malignancy, and new immunotherapeutic approaches are actively being tested. In relapsed and refractory MM patients, an early clinical trial supported good efficacy (ORR 44%) with an acceptable safety profile of pembrolizumab, lenalidomide (an immunomodulatory imide drug: IMiD), and low dose dexamethasone [56]. However, the combination of pembrolizumab and IMiD showed unfavorable benefit-risk profile in following phase III trials in relapsed and refractory MM (KEYNOTE-183) and newly diagnosed MM (KEYNOTE-185) [57, 58].

It remains poorly understood why PD-1 blockade failed to show clinical benefits in MM patients. As MM predominantly grows in the bone marrow (BM), the unique TME in the MM BM might be implicated in therapeutic resistance [95]. One of the key factors is an altered T cell phenotype in the MM BM. In general, T cell subsets with a senescent phenotype represent dysfunctional T cells in the TME and often result in hypo-responsiveness to ICB therapy [96]. A recent scRNA-seq analysis of the MM immune microenvironment showed that a senescent T cell subset emerges in a premalignant state (i.e., smoldering MM) [97], indicating that symptomatic MM patients already have dysfunctional T cells at diagnosis. Moreover, dysfunctional T cells are increased in patients who experience relapse after autologous SCT [98] indicating that there is difficulty in reinvigorating T cells in relapsed and refractory MM patients. Another key barrier for anti-tumor immunity is immunosuppressive subsets in the TME. As myeloid cells and their progenitors abundantly exist in the BM, MM progression triggers conversion of these cells into myeloid-derived suppressor cells (MDSCs). Indeed, the transcriptional landscape in MM patients revealed an inverse correlation between MDSC-related genes and cytotoxic lymphocyte-related genes suggesting that MDSCs might contribute to T cell exclusion in the MM BM [99]. MM-associated Tregs are important targetable immunosuppressive cells since CD38^+^ Tregs can be depleted by daratumumab (anti-CD38 mAb) [100]. However, a phase 1 clinical trial of daratumumab in combination with anti-PD-1 mAb (JNJ-63723283) in relapsed and refractory MM (MMY2036, NCT03357952) was terminated due to limited clinical responses with increased adverse events in the combination group compared to daratumumab monotherapy.

While PD-1 blockade has not demonstrated clinical benefits in MM patients, it is noteworthy that some patients achieved long-term remissions after stopping pembrolizumab in clinical trials [101]. Thus, further investigations are necessary to predict responder patients. Alternatively, T cell immunoreceptor with Ig and ITIM domains (TIGIT) has emerged as an important immune checkpoint on MM T cells [102]. The inhibitory mechanisms of this new immune checkpoint will be discussed later in this review.

### Myeloid malignancies

Early phase 1 trials showed lack of efficacy of anti-PD-1 mAb as monotherapy in patients with acute myeloid leukemia (AML) [103] or high-risk myelodysplastic syndrome (MDS) [104]. Similarly, CTLA-4 blockade using ipilimumab failed to show clinical benefits in high-risk MDS patients [105].

Although these early trial results are disappointing, ipilimumab treatment after allogeneic-hematopoietic stem cell transplantation (HSCT) showed good responses in 22 patients with various hematological malignancies including 12 AML patients (CR 23% in 4 patients with extramedullary AML and 1 with MDS developing into AML) [59], suggesting that ipilimumab can augment graft-versus-leukemia (GvL) effects. Of course, the key concern remains to be immune-related adverse events (irAEs), especially graft-versus-host disease (GVHD). However, among 22 patients, 3 patients in total developed GVHD (1 patient with grade II acute GVHD of the gut and 2 patients with chronic GVHD of the liver) which was able to be controlled by glucocorticoids [59]. PD-1 blockade after allogeneic HSCT is also being tested, but so far, it has shown modest clinical benefits [106].

Another potential approach to improve ICB efficacy is using a hypomethylating agent (HMA) such as azacytidine. Azacytidine is clinically approved for MDS and AML and has shown immunomodulatory effects including upregulation of MHC-I. In a recent trial, the combination of nivolumab and azacytidine showed an ORR of 33% and CR rate of 22% in 70 patients with relapse/refractory AML [60]. Intriguingly, while high infiltration of effector T cells before therapy predicted good responsiveness to the combination therapy, an increase in CTLA-4^+^ effector lymphocytes after treatment predicted non-responder patients [60]. The combination of HMAs and PD-1 blockade are currently being tested in several trials [106], but co-blockade of PD-1 and CTLA-4 may potentially overcome therapeutic resistance.

Immune dysregulation in AML remains incompletely understood as it might be differentially organized and depends on multiple factors such as tumor subtypes, presence or absence of the MDS state, patients’ age, and treatment history. Kong et al. originally showed high TIGIT expression on CD8^+^ T cells in AML patients, and that siRNA mediated TIGIT knockdown can reinvigorate cytokine production suggesting that TIGIT is expressed on dysfunctional T cells [107]. Another independent group also showed that PD-1^+^ TIGIT^+^ CD8^+^ T cells with low expression levels of DNAM-1 (DNAX accessory molecule 1 or CD226) represent dysfunctional T cells [108], further providing evidence that TIGIT represents a key checkpoint molecule in AML. In terms of mechanisms of immune escape after HSCT, Christopher et al. recently performed exome sequencing on paired samples obtained at diagnosis and at post-HSCT relapse demonstrating that downregulation of MHC II-related genes is a key feature of relapsed AML [109]. This result suggests that CD4^+^ T cells might play a critical role for controlling relapse. Thus, it might be possible to augment CD4-mediated GvL effects by blockade of LAG-3-MHC-II interaction. Another immune checkpoint TIM-3 is also expressed on AML T cells [110], but tumor TIM-3 has a unique tumor-intrinsic role, as discussed later. Thus, TIGIT, LAG-3, and TIM-3 are differentially implicated in AML immune-regulation and are rational therapeutic targets in combination with PD-1 blockade and/or CTLA-4 blockade.

Compared to MDS and AML, therapeutic benefits of ICB may be better achieved in myeloproliferative neoplasms (MPNs), a heterogeneous group of diseases characterized by clonal expansion of myeloid cells. Significant advances have been made to control disease progression by targeting JAK2, one of the key driver mutations in MPNs. However, it is still challenging to treat high-risk patients who are refractory to JAK1/JAK2 inhibitors [111]. Prestipino et al. showed that JAK2 mutations can trigger the overexpression of PD-L1 on myeloid cells, leading to metabolic and functional impairment of T cells [112]. Importantly, PD-1 blockade improved survival in human MPN xenograft and primary murine MPN models [112] suggesting potential roles for immunotherapy in MPNs. Another notable aspect is immunogenicity of MPNs. The JAK2V617F mutation is seen in > 50% of patients with MPNs, and this mutation-associated neoantigen can elicit tumor-specific CD8^+^ T cell responses [113]. Somatic frameshift mutation of the calreticulin gene (*CALR*) is another MPN-restricted key driver mutation found in 67% and 88% of patients with essential thrombocythemia and primary myelofibrosis respectively [114]. These *CALR* mutations induce a shared MPN-specific neoantigen leading to the generation of mutant calreticulin–specific T cell responses [115, 116]. Thus, due to the unique immunogenicity and induction of neoantigen-specific T cell responses, ICB might play an important role for disease control in MPNs. However, further clinical investigations are warranted.

## Beyond PD-1 and CTLA-4 blockade

**Fig. 3:**
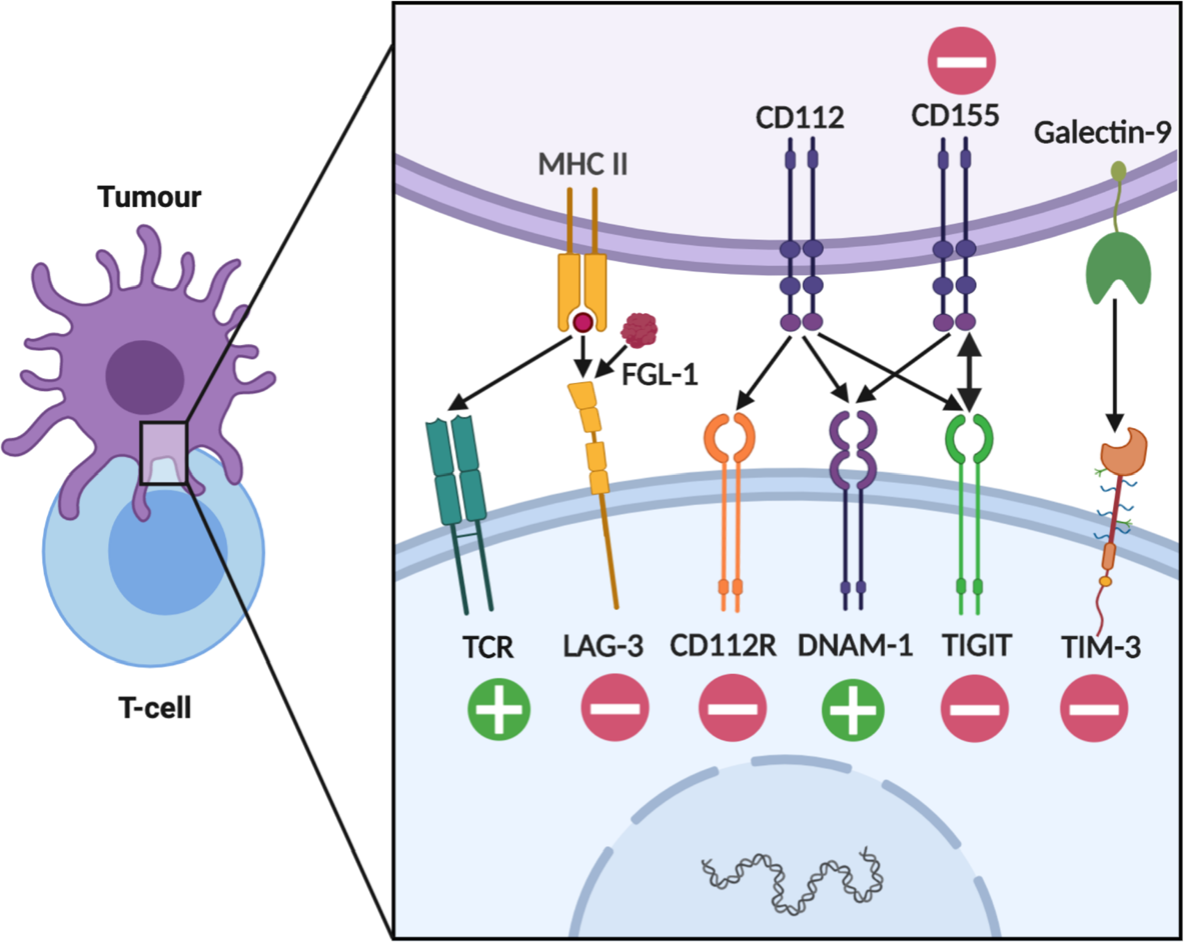
Negative regulators of T cell immunity other than PD-1 and CTLA-4. Schematic illustrating receptors and their ligands regulating T cell immunity. Multiple immune checkpoint molecules are differentially implicated in the regulation of activated T cells including LAG-3, TIM-3, and TIGIT. Plus and minus signs denote stimulatory and inhibitory signaling respectively. Single-headed and double-headed arrows denote uni-directional and bi-directional signaling respectively. APC, antigen-presenting cell; TCR, T cell receptor; LAG-3, lymphocyte-activation gene 3; CD112R, CD112 receptor; MHC, major histocompatibility complex; FGL1, fibrinogen-like protein 1; DNAM-1, DNAX accessory molecule 1; TIGIT, T cell immunoreceptor with Ig and ITIM domains; TIM-3, T cell immunoglobulin mucin-3

Therapeutic blockade of PD-1/PD-L1 or CTLA-4 has shown clinical benefits in only certain types of hematological malignancies such as HL. Obviously, inhibiting different immune checkpoint molecules on T cells (such as LAG-3, TIM-3, and TIGIT) can be a potential approach since these molecules inhibit T cell responses in a non-redundant manner (Fig. 3).

### LAG-3

Lymphocyte-activation gene 3 (LAG-3, CD223) is a transmembrane protein reported to be primarily expressed on tumor-infiltrating T cells. It consists of four extracellular immunoglobulin (Ig)-like domains (D1–D4) with high homology to CD4 [117], and it binds to complexes of peptides and MHC-II [118].

Although the impacts of LAG-3-MHC-II interaction on CD4^+^ T cell immunity have been well studied, it was poorly understood as to how LAG-3 negatively regulates CD8^+^ T cell-mediated anti-tumor immunity [119, 120]. Recently, Wang et al. identified fibrinogen-like protein 1 (FGL1) as a high affinity soluble ligand for LAG-3 and reported that FGL1-LAG3 interaction critically impedes CD8^+^ T cell-mediated anti-tumor immunity [121]. While FGL-1 is upregulated in solid tumor tissues such as lung, prostate, and breast cancer [121], it remains unknown whether hematological malignancies also produce abundant FGL-1. It should be noted that unlike solid tumor cells, hematological tumor cells frequently express MHC-II, and thus, the LAG-3-MHC-II interaction still plays a key inhibitory role in CD4^+^ T cell-mediated control. As LAG-3 is frequently co-expressed with PD-1 in tumor infiltrating lymphocytes, combined efficacy of PD-1 and LAG-3 blockade (NCT03005782 and NCT02061761) or a bispecific antibody against PD-1 and LAG-3 (NCT03219268) are actively being investigated in various types of malignancies including blood cancers. More recently, Keane et al. investigated LAG-3 expression in DLBCL patients. Indeed, LAG-3 was co-expressed with PD-1 on Tregs and CD8^+^ T cells; however, strikingly, LAG-3 expression was also observed on tumor-associated macrophages and a proportion of malignant B cells [122]. In addition to the clinical efficacy of dual PD-1 and LAG-3 blockade, functional impacts of myeloid LAG-3 and tumor-intrinsic LAG-3 will require further investigation.

### Tim-3

TIM-3 (T cell immunoglobulin and mucin domain 3) is often co-expressed with PD-1 on tumor infiltrating T cells and has been recognized as a potential target for combination blockade with PD-1 [123–125]. TIM-3 signaling can induce tolerance in T cells upon recognition of its major ligand (galectin-9) [126] that is widely expressed in various types of malignancies including blood cancers. While TIM-3 acts as one of the T cell immune checkpoint molecules, TIM-3 on non-lymphoid cells is also implicated in immune-regulation. In preclinical breast cancer models treated with paclitaxel and anti-TIM-3 mAb, TIM-3^+^ intra-tumor CD103^+^ dendritic cells (DCs) are a key target subset that can mobilize CD8^+^ T cells via CXCL9 production in response to the combination therapy [127]. Additionally, by analyzing tumor tissues from NHL patients, Huang et al. showed that TIM-3 is highly expressed on endothelial cells (ECs) in the lymphoma microenvironment. Importantly, TIM-3 on ECs inhibits CD4^+^ T cell-mediated immunity, and thus, high expression levels of TIM-3 on the endothelium are associated with advanced stage and higher international prognosis index scores [128]. Another notable role of TIM-3 is its tumor-intrinsic function in AML. TIM-3 is one of the markers highly expressed on leukemia stem cells [129], and tumor-derived galectin-9 promotes self-renewal in an autocrine manner [130]. Since TIM-3 is highly expressed on T cells in AML patients [110, 131], TIM-3 blockade might have multiple therapeutic benefits in AML. In a phase 1 clinical trial of anti-TIM-3 mAb with or without anti-PD-L1 mAb in patients with advanced cancers, both monotherapy and combination therapy showed a good safety profile [132]. Given the broad expression of TIM-3 on innate and adaptive immune cells, endothelial cells, and certain types of tumor cells, it is possible that TIM-3 blockade might have pleiotropic effects. Further studies are warranted to understand the expression and function of TIM-3 in disease-specific tumor microenvironments.

### TIGIT

An activating receptor DNAM-1 (CD226) on cytotoxic lymphocytes plays a critical role for recognition and elimination of tumor cells by recognition of its ligand CD155 and CD112. Its inhibitory counterparts, TIGIT and CD96, also share CD155 as a ligand, and thus, these inhibitory receptors negatively regulate DNAM-1-dependent functions [133]. CD112, on the other hand, can also bind to the co-inhibitory CD112 receptor expressed on T cells where it can compete with CD226 binding to CD112 [134]. Among these receptors, TIGIT has the highest binding affinity to CD155. Of note, TIGIT-mediated inhibitory functions are explained by multiple mechanisms beyond competition with DNAM-1 for CD155 binding. At the interface between T cells and APCs, the TIGIT-CD155 interaction can bi-directionally inhibit T cells and APCs [135]. Moreover, TIGIT^+^ Tregs potently suppress anti-tumor immunity [136]. Although crucial inhibitory mechanisms remain unknown, growing evidence suggests that TIGIT is a key T cell immune checkpoint in hematological malignancies. As described earlier, DNAM-1^low^PD-1^+^TIGIT^+^ subset represents dysfunctional T cells in AML patients, and an increased frequency of this subset predicts poor prognosis in AML patients [108]. In MM, TIGIT is the most frequently upregulated checkpoint molecule among PD-1, CTLA-4, LAG-3, and TIM-3, and TIGIT^+^ T cells represent a dysfunctional T cell subset [102]. In a mouse Vk*MYC MM model, DNAM-1^low^PD-1^+^TIGIT^+^ dysfunctional T cells are increased at post-transplant relapse [137] suggesting that the emergence of dysfunctional T cells might be tightly associated with immune escape. Additionally, TIGIT also negatively regulates NK cell-dependent control of tumors [138]. Since CD155 is expressed in a broad range of malignancies including blood cancers, therapeutic blockade of TIGIT might have broad implications for immunotherapy against hematological malignancies.

## Innate checkpoints

**Fig. 4:**
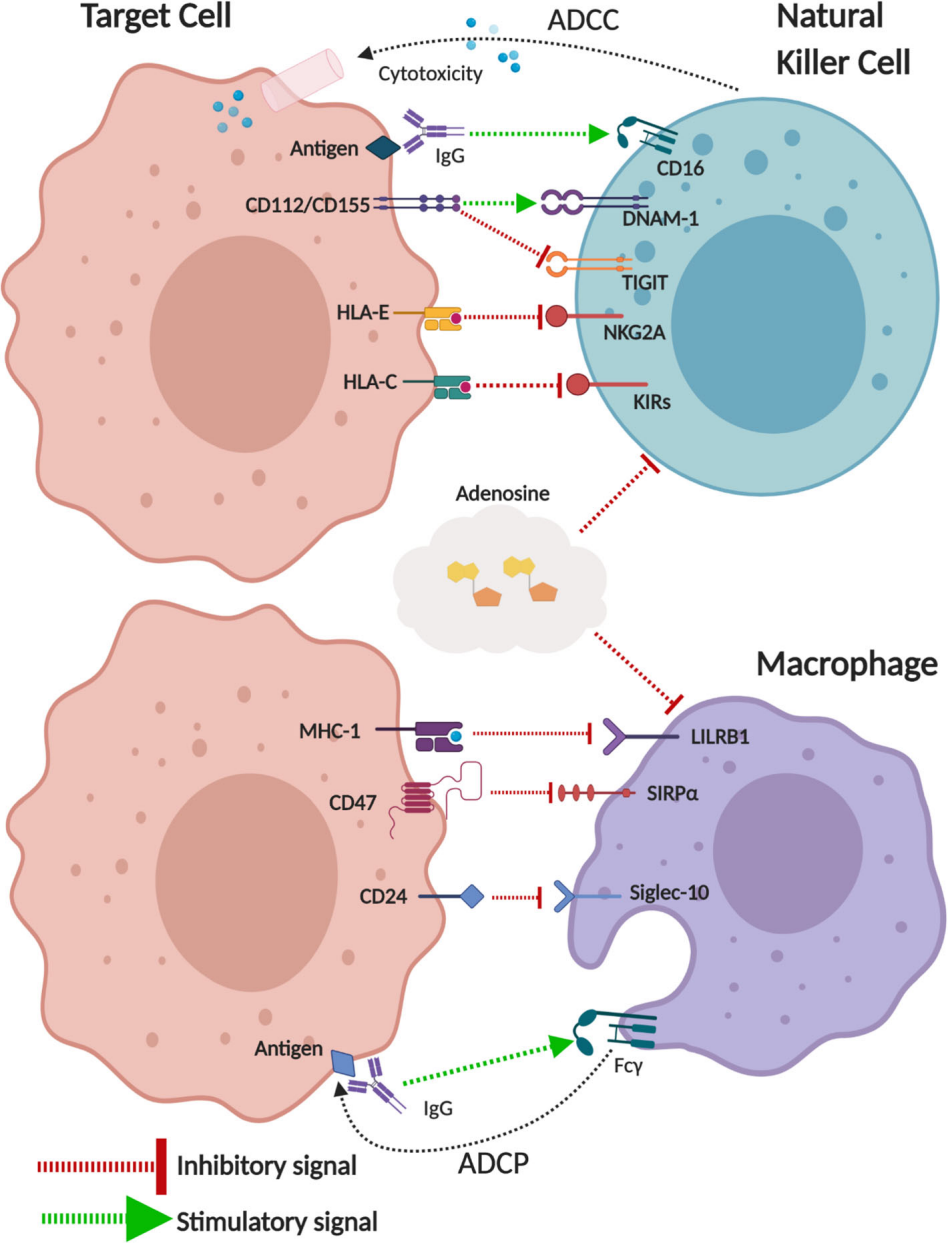
Negative regulators of innate anti-tumor immunity. Schematic illustrating receptors and their ligands regulating anti-tumor immunity by NK cells (top) and macrophages (bottom). In NK cells, inhibitory receptors that recognize MHC class 1 molecules are recognized as a potential target to enhance NK cell-mediated cytotoxicity against tumors. Targeting macrophage phagocytosis checkpoints has also emerged as a potential approach in combination with various cancer mAb therapies due to its potential in enhancing the elimination of antibody-coated tumor cells. An immunosuppressive metabolite, adenosine, also potently inhibits innate and adaptive anti-tumor immunity. ADCP, antibody-dependent cellular phagocytosis; ADCC, antibody-dependent cellular cytotoxicity; DNAM-1, DNAX accessory molecule 1; TIGIT, T cell immunoreceptor with Ig and ITIM domains; NKG2A, NK group 2 member A; KIRs, killer-cell immunoglobulin-like receptors; HLA, human leukocyte antigen; MHC, major histocompatibility complex; LILRB1, leukocyte immunoglobulin-like receptor B1; SIRPα, signal regulatory protein α, Siglec-10, Sialic acid-binding Ig-like lectin 10

ICB therapies such as anti-PD-1 and anti-CTLA-4 predominantly depend on T cells for their therapeutic efficacy. However, multiple factors are implicated in primary and acquired resistance including (1) low TMB and neoantigen load, (2) low infiltration of tumor-specific T cells, and (3) loss of β2M/MHC-I. In this light, harnessing innate anti-tumor immunity by NK cells [139] or macrophages [140] may be a potential approach (Fig. 4).

### NK cell inhibitory checkpoints

The activation of NK cells is regulated by a balance between activation receptor signaling and inhibitory receptor signaling. While activation receptors such as NKG2D (natural-killer group 2, member D) and DNAM-1 recognize ligands upregulated on malignantly transformed cells, inhibitory receptors such as NKG2A (NK group 2 member A) and KIRs (killer cell immunoglobulin-like receptors) recognize non-classical MHC-I (HLA-E) and classical MHC-I, respectively. Thus, NK cells can eliminate transformed cells that express self-induced danger ligands “induced-self recognition” or abnormal cells that lose MHC-I expression “missing-self recognition” [141].

NKG2A is expressed on both NK cells and T cells, and its ligand HLA-E is frequently overexpressed in various malignancies including DLBCL, MM, and AML [142, 143] which allows tumor cells to evade from cytotoxicity. Indeed, an early clinical study demonstrated that anti-NKG2A mAb (monalizumab) in combination with cetuximab (anti-EGFR mAb) augments therapeutic efficacy by enhancing ADCC in head and neck cancer patients [144]. The therapeutic potential of NKG2A blockade in combination with various mAbs with ADCC activity requires further investigation in hematological malignancies.

IPH2101 (1-7F9) is an anti-KIR mAb that inhibits the interaction between KIRs (KIR2DL-1, KIR2DL-2, and KIR2DL-3) and their HLA-C ligands. It showed a good safety profile in phase I clinical trials [145, 146]; however, a subsequent phase II trial in smoldering MM patients showed limited clinical benefits as monotherapy [147]. The combination of IPH2101 and lenalidomide showed some responses in relapsed and refractory MM [148]. Currently, the recombinant version with a stabilized hinge, lirilumab (IPH2102/BMS-986015), is being tested in various malignancies. Recently, a combination of lirilimumab and nivolumab was trialed in a small number of patients with relapsed or refractory lymphoid malignancies. The combination was unable to improve on the already strong therapeutic efficacy of nivolumab in cHL, and no therapeutic benefits of the combination were recorded in an unselected cohort of NHL or MM patients [149].

### Macrophage phagocytosis checkpoints

Tumor-associated macrophages (TAMs) are educated to promote tumor growth and immunosuppression, and their abundance is often correlated with poor prognosis in various types of malignancies including blood cancers [140]. However, TAMs critically contribute to the therapeutic efficacy of cancer mAb-based therapies by their Fc receptor-dependent ADCP activity. In this context, several negative regulators of ADCP have been identified, and blockade of “don’t eat me signals” has emerged as a new approach to augment engulfment and clearance of tumor cells by monocytes/macrophages [150].

The interaction between signal regulatory protein α (SIRPα) and its ligand CD47 is one of the best characterized “don’t eat me” signals. The SIRPα receptor contains an immunoreceptor tyrosine-based inhibitory motif (ITIM), and upon CD47 recognition, this receptor transmits negative signals that attenuate phagocytic activity [151]. While its ligand, CD47, is expressed on various normal cell types, it is often found to be overexpressed in malignant tumor cells. Indeed, growing preclinical evidence supports that CD47 blockade augments lymphoma cell clearance either as monotherapy [152] or in combination with rituximab [153]. Of note, CD47 blockade can also induce adaptive immunity by enhancing DC-mediated cross-priming [152] suggesting that the combination of CD47 blockade with ICB might also be a rational approach. In a recent clinical trial, anti-CD47 mAb (Hu5F9-G4) in combination with rituximab has shown good clinical responses in relapsed and refractory NHL with a CR of 43% (in FL patients) and 33% (in DLBCL patients) [154]. Given that these patients were heavily pretreated, the efficacy of the combination is promising. Importantly, on-target adverse events such as anemia (due to phagocytosis of erythrocytes) were manageable and transient which support its safety profile [154]. Currently, several CD47 blocking agents are actively being tested in clinical trials in various types of hematological malignancies. More recently, other mediators for “don’t eat me signals” have been identified such as the interaction of LILRB1-MHC-I [155], Siglec-10-CD24 [156], and immunosuppressive adenosine signaling [157]. While their redundant and non-redundant roles require further investigation, these phagocytosis regulators are promising targets for enhancing ADCP against hematological malignancies.

## ICB and CAR T cell therapy

Chimeric antigen receptor (CAR) T cells are synthetically engineered T cells expressing a CAR that has the target specificity to bind an antigen in an MHC-independent manner [158]. Encouraging results from recent trials with anti-CD19 CAR T cell therapies lead to their FDA approval for treatment of relapsed or refractory B cell acute lymphoblastic leukemia (ALL) and adult large B cell lymphoma [159–165].

Despite encouraging outcomes of anti-CD19 CAR T cell therapies, poor T cell persistence remains to be a major reason for relapse or a lack of response after CAR T cell therapy. Indeed, only 29% of the CLL patients had a CR to anti-CD19 CAR T cell therapy [166] in contrast to the 90% CR rate reported in ALL patients [167]. Notably, non-responder CLL patients showed transcriptional upregulation of genes related to apoptosis and exhaustion while responder patients had lower proportions of PD-1-expressing CAR T cells [168]. Moreover, co-expression of PD-1 with LAG-3 or TIM-3 on CAR T cells were associated with poor responses [168], highlighting the critical role of T cell immune checkpoint molecules for limiting CAR T cell activity. Still, clinical benefits of combining PD-1/PD-L1 inhibitors with CD19 CAR T cell therapy are yet to be determined though this combination is thus far reported to have an acceptable safety profile [169, 170]. Another recent trial of CD19 CAR T cell therapy in combination with PD-1 blockade in 14 children with heavily pre-treated B-ALL provided further evidence of the safety profile of this combination therapy. In fact, 3 of 6 patients that received CAR T cell therapy with a PD-1 inhibitor re-established B cell aplasia, suggesting that PD-1 blockade may prolong CAR T cell activity [171].

Alternatively, genetic modification of CAR T cells is another potential approach to augment the efficacy of CAR T cell therapy. Various “Armoured” CAR T cells have been generated to express immuno-stimulatory ligands and cytokines such as CD40 ligand [172], Fms-like tyrosine kinase 3 ligand (Flt3L) [173], IL-12 [174, 175], and IL-18 [176]. To inhibit CAR T cell-intrinsic PD-1, shRNA or engineering PD-1 dominant negative receptors have been developed [177]. Additionally, genome editing techniques such as CRISPR/Cas9 [178] as well as TALEN [179] have also been used to delete PD-1, and PD-1-deficient CAR T cells have shown improved cytotoxicity in vivo [178]. However, off-target cleavage and guide-RNA associated genotoxicity should be thoroughly interrogated prior to clinical applications. More recently, Rafiq et al. developed modified CAR T cells that secrete PD-1-blocking single-chain variable fragments (scFv). Notably, this approach has demonstrated improved anti-tumor responses in addition to mobilizing the bystander tumor-specific T cells in solid and hematological malignancies [180]. It is possible that the smaller size of scFvs locally released in the TME may help reduce the risk of irAEs compared to systemic ICB therapies.

Possibly, combination with inhibitory molecules such as TIM-3 and LAG-3, in addition to PD-1 blockade, may further improve CAR T cell function though much remains to be studied to support the proof-of-concept. Given the high incidence of cytokine release syndrome associated with CAR T cell therapies [181], safety concerns remain a major barrier to simultaneously target multiple immune checkpoint molecules in combination. Nonetheless, safety strategies that can limit exacerbation of toxicities should be considered such as the suicide gene “safety switch” systems, including iCaspase-9, already employed in clinical trials [182]. Efficient manufacturing of CAR T cell products remains another major issue in patients with highly proliferative circulating leukemic blasts with relatively fewer T cells available [183]. To this end, off-the-shelf, allogeneic CAR T cells or induced pluripotent stem cell (iPSC)–derived CAR T products hold great potential to overcome problems associated with CAR T cell production. Overall, advances in genome editing as well as manufacturing processes may lead to potentially curative therapies for patients with hematological malignancies.

## Concluding remarks

Although ICB therapies, particularly PD-1/PD-L1 blockade, are being actively tested in a number of hematological malignancies, it remains to be fully understood why blockade of PD-1/PD-L1 shows efficacy in only limited tumor types. The immunologically “cold” TME might act as a major barrier for therapeutic blockade of T cell checkpoints, and combining ICB with chimeric antigen receptor (CAR) T cell therapy or bispecific T cell engagers is an active area of investigation [184, 185]. Indeed, irAEs are a major concern since cytokine-release syndrome is frequently observed in patients treated with T cell-based approaches. In addition to primary resistance, acquired resistance mechanisms of ICB remain to be elucidated. Even in patients with HL, most eventually experience disease progression during anti-PD1 therapy [186]. Targeting other T cell checkpoint molecules may be a potential approach to overcome acquired resistance. However, an in-depth characterization of T cell function and checkpoint molecules will be highly warranted to determine the optimal therapeutic approach. Harnessing innate anti-tumor immunity by NK cells and macrophages could be another rational approach against tumors with downregulated MHC-I expression. Again, a comprehensive understanding of the immune microenvironment will provide a clue for identifying biomarkers that can predict responsiveness to ICB. Alternatively, a personalized approach may be necessary given that the disease-specific immune microenvironment can be sculpted by multiple factors including tumor genotypes, treatment history, and comorbidities in patients. Therefore, a mechanistic understanding of the role of tumor intrinsic and extrinsic modulators of therapeutic response will hopefully inform avenues for durable disease control from ICB with minimal immune-related toxicities.

## Data Availability

Not applicable

## Abbreviations

ADCC: Antibody-dependent cellular cytotoxicity
ADCP: Antibody-dependent cellular phagocytosis
AML: Acute myeloid leukemia
APC: Antigen presenting cells
BM: Bone marrow
CALR: Calreticulin
CAR: Chimeric antigen receptor
CR: Complete response
CTLA-4: Cytotoxic T lymphocyte-associated protein 4
DCs: Dendritic cells
DLBCL: Diffuse large B cell lymphoma
DNAM-1: DNAX accessory molecule 1
FGL-1: Fibrinogen-like protein 1
FL: Follicular lymphoma
GVHD: Graft-versus-host disease
GvL: Graft-versus-leukemia
HL: Hodgkin lymphoma
HLA: Human leukocyte antigen
HMA: Hypomethylating agent
HRS: Hodgkin Reed-Sternberg
HSCT: Hematopoietic stem cell transplantation
ICB: Immune checkpoint blockade
IMiDs: Immunomodulatory imide drugs
IPEX: Immunodysregulation polyendocrinopathy enteropathy X-linked
irAEs: Immune-related adverse events
ITIM: Immunoreceptor tyrosine-based inhibitory motif
KIRs: Killer cell immunoglobulin-like receptors
LAG-3: Lymphocyte-activation gene 3
LILRB1: Leukocyte immunoglobulin-like receptor B1
LRBA: Lipopolysaccharide-responsive and beige-like anchor protein
mAb: Monoclonal antibodies
MDS: Myelodysplastic syndrome
MDSCs: Myeloid-derived suppressor cells
MM: Multiple myeloma
MPNs: Myeloproliferative neoplasms
NHL: Non-Hodgkin lymphoma
NK cell: Natural killer cell
NKG2A: NK group 2 member A
ORR: Objective response rate
PD-1: Programmed cell death protein 1
PD-L1: Programmed death ligand 1
scRNA-seq: Single-cell RNA sequencing
SCT: Stem cell transplant
Siglec-10: Sialic acid-binding Ig-like lectin 10
SIRPα: Signal-regulatory protein α
TAMs: Tumor-associated macrophages
TCR: T cell receptor
TIGIT: T cell immunoreceptor with Ig and ITIM domains
TIM-3: T cell immunoglobulin and mucin domain 3
TMB: Tumor mutation burden
TME: Tumor microenvironment
Tregs: Regulatory T cells
β2M: β2-microglobulin
